# Sonic Hedgehog Signaling: Evidence for Its Protective Role in Endotoxin Induced Acute Lung Injury in Mouse Model

**DOI:** 10.1371/journal.pone.0140886

**Published:** 2015-11-06

**Authors:** Xing Chen, Yuting Jin, Xiaoming Hou, Fengqin Liu, Yulin Wang

**Affiliations:** 1 Department of Pediatrics, Shandong Provincial Hospital Affiliated to Shandong University, Jinan, Shandong, The People’s Republic of China; 2 Department of Pediatrics, Shandong Provincial Qianfoshan Hospital, Jinan, Shandong, The People’s Republic of China; Indiana University School of Medicine, UNITED STATES

## Abstract

**Objective:**

To investigate the protective role of the sonic hedgehog (SHH) signaling associated with a lipopolysaccharide (LPS)-induced acute lung injury (ALI) in a mouse model.

**Methods:**

Male BALB/c mice were randomly divided into four groups: control, LPS, LPS-cyclopamine group and cyclopamine group. ALI was induced by LPS ip injection (5 mg/kg). The sonic hedgehog inhibitor cyclopamine (50 mg/kg) was given to the LPS-cyclopamine group at 30 min after LPS injection as well as normal mice as control. Lung injury was observed histologically in hematoxylin and eosin (HE) stained tissue sections, semi-quantified by lung tissue injury score, and the lung tissue mass alteration was measured by wet to dry weight ratio (W/D). mRNA expression levels of TNF-α, SHH, Patched (PTC) and GLI1 in lung tissue were studied with real time quantitative PCR (RT-PCR), while the protein expression of SHH and GLI1 was determined by western blot analysis.

**Results:**

Lung tissue injury score, thickness of alveolar septa, W/D, and TNF-α mRNA expression levels were significantly higher in the ALI mice than the normal mice (P<0.05). The mRNA expression levels of SHH, PTC, and GLI1 in the ALI mice were significantly higher at 12h and 24h after LPS injection, but not at the 6h time point. Protein production of SHH and GLI1 at 6h, 12h, and 24h in the lungs of ALI mice significantly increased, in a time-dependent manner, compared with that in normal mice. Cyclopamine alone has no effect on pathological changes in normal mice. Intervention with cyclopamine in ALI mice led to a reduction in mRNA levels of SHH, PTC, and GLI1 as well as SHH and GLI1 protein levels; meanwhile, the pathological injury scores of lung tissues, thickness of alveolar septa, W/D, and mRNA expression levels of TNF-α increased compared with mice receiving LPS only.

**Conclusion:**

The SHH signaling pathway was activated in response to LPS-induced ALI, and up-regulation of SHH expression could alleviate lung injury and be involved in the repair of injured lung tissue.

## Introduction

Acute lung injury (ALI), a milder form of acute respiratory distress syndrome (ARDS), is a condition characterized by acute severe hypoxia that is caused by onset of pulmonary inflammation due to infection, shock, trauma and burns, as well as other non-cardiac disease [1]. Infection and systemic inflammatory response lead to diffuse alveolar damage, alveolar capillary leakage, severe hypoxemia, and poor lung compliance. This complication often requires intensive care, and the morbidity and mortality is high. The mortality rate remains as high as 25% to 50% when ALI is caused by serious Gram negative bacterial infections [2]. Despite great efforts has been made to investigate pathogenesis, treatment, and prevention, current knowledge is still far from completely understanding the mechanism of the syndrome.

The sonic hedgehog (SHH) signaling pathway is an important signal transduction pathway for the formation of embryonic lung structure and its developmental processes [3,4]. The SHH pathway consists mainly of SHH protein, transmembrane receptor Patched (PTC), Smoothened (SMO), and the downstream GLI1 transcription factor. In the absence of SHH protein, PTC interacts with SMO and inhibits it from being active, therefore blocking signaling. When SHH protein is present, it interacts with PTC and leads to derepression of SMO, which results in activation of downstream transcription factor GLI1 [5]. The activity of the pathway is greatly reduced after birth and in adult tissues [6,7], but is reactivated in some lung diseases, including pulmonary hypoplasia, interstitial lung disease, chronic obstructive pulmonary disease (COPD), asthma, and lung cancer [8,9,10,11,12,13,14,15,16]. However, the role of the SHH signaling pathway in endotoxin induced ALI remains unclear.

Lipopolysaccharide (LPS), also known as endotoxin, is one of the most well known agents that induce a strong inflammatory response in both humans and animals. The LPS induced ALI in the mouse model reflects some of the important pathologic processes of human ALI, such as the increase of vascular permeability and neutrophil infiltration [17]. LPS binds to the CD14/TLR4 receptor complex on the surface of monocytes/macrophages and endothelial cells, promotes the production and release of inflammatory cytokines including TNF-α, IL-1β, IL-6, and IL-8 from these cells [18]. TNF-α is one of the most important inflammatory cytokines, known as "early response cytokines," which contributes to the systemic or local inflammatory reaction that results in ALI and ARDS.

In this study, we investigated the function of the SHH pathway in the response of ALI by using a LPS-induced mouse ALI model. Our results supported an important role for SHH in protecting lung tissue from further damage through anti-inflammatory responses.

## Materials and Methods

### ALI animal model

The animal experimental protocol was approved by the Institute of Animal Care and Use Committee of the Medical School of Shandong University. The ALI mouse model was established with male BALB/c mice (5 to 7 weeks old, 20-25g in body weight) purchased from the Experimental Animal Center of Shandong University. Thirty mice were injected intraperitoneally (ip) with LPS (Sigma-Aldrich, St. Leuis, USA) at 5 mg/kg to establish ALI. For the control mouse group, fifteen mice were injected ip with the same volume of PBS or cyclopamine (50 mg/kg, Sigma-Aldrich, St. Louis, USA). Fifteen of the established ALI mice were further treated with a cyclopamine injection ip at 50mg/kg 30 min after LPS injection, designated as the LPS-C group. At 6h, 12h, or 24h after the final injection, five animals from each group were sacrificed by CO_2_ asphyxiation and cervical dislocation, and the lungs were harvested for study. The left upper lobe of the harvested lung was fixed for histology, the left lower lobe was used immediately for the wet to dry weight ratio (W/D) ratio determination, and the right lung was flash frozen in liquid nitrogen and stored for RNA and protein analysis.

### Histopathological examination

The left upper lobe of the lung was fixed in 4% paraformaldehyde at 4°C overnight, embedded in paraffin wax, and sectioned for HE staining. Histopathological examination of the sections was performed by measuring the thickness of the alveolar septa as well as semi-quantitative scoring, as described earlier [19], for 5 different aspects: pulmonary edema, inflammatory infiltration, hemorrhage, atelectasis and hyaline membrane formation: 0 for no injury, 1 for injury < 25%, 2 for injury ranging from 25% to 50%, 3 for injury ranging from 50% to 75%, and 4 for injury > 75%. Ten high magnitude fields from each slide were analyzed, and data are shown as mean±SEM (standard error of the mean).

Lung tissue mass alteration was measured with the left lower lobe of the lung from each mouse. The lung tissue was dried with filter paper, weighed, and baked at 60°C for 48 h and weighed again. The W/D was calculated.

### RT-PCR analysis

Total RNA was extracted with TRIzol (TaKaRa, IL USA) from the frozen sample of the right lung, and reverse transcribed into cDNA with PrimeScript RT reagent Kit (TaKaRa, IL USA). Then RT-PCR was performed for each target mRNA with corresponding primers listed in **S1 Table** and SYBR® Premix Ex Taq (TaKaRa, IL USA) reaction system in a total volume of 20 μL. Reaction conditions were set as: 95°C 30 s, 1 cycle; 95°C 5 s, 60°C 30 s, 40 cycles; 95°C 5 s, 60°C 1 min, 50°C 30 s, 1 cycle. β-actin was used as an internal control. Each testing was performed in triplicate tubes and repeated three times.

### Western blot analysis

Total protein was extracted from the frozen sample of the right lung and protein expression was assessed by Western blot. Protein samples were separated by SDS- polyacrylamide gel electrophoresis (SDS-PAGE), and transferred to PVDF membrane. The membrane was blocked in 5% nonfat dry milk for 1h at room temperature prior to primary antibody (Abcam, MA, USA, 1:500 dilution of rabbit anti-mouse SHH antibody; 1:300 dilution of rabbit anti-mouse GLI1 antibody) incubation at 4°C overnight. Incubation with horseradish peroxidase conjugated goat anti-rabbit secondary antibody (1:5000, Zhongshan, Beijing, China) was performed at room temperature for 1h. Immune-reactive signals were detected using an ECL kit. The blots were also probed with an anti-β-actin antibody (Sigma) as loading control and internal reference. A semi-quantitative analysis was performed with Multi Gauge V3.2 software for the ratio of absorbance values of target protein band to β-actin internal reference.

### Statistical analysis

Statistical analysis was performed using SPSS 17.0 statistical analysis (IBM, USA), and data were expressed as mean ± SEM. Differences between the means of two groups were determined by one-way ANOVA. Unpaired t-tests were used to assess the statistical differences between the groups. Two tailed P <0.05 was considered statistically significant.

## Results

### Pathological observations

An ALI animal model was successfully established by a single ip injection of LPS to BALB/c mice. Lungs of the LPS-injected mice showed significant swelling on the surface and were dark red in color with diffused bleeding spots. The tissue swelling and bleeding was further aggravated by cyclopamine administration. Mice injected with LPS plus cyclopamine (LPS-C) demonstrated severely swollen lung tissue deep dark red in color with large-scale bleeding and petechiae spots. Visual observation of lung tissue from the PBS and cyclopamine injected mice found no obvious abnormalities.

**Fig 1:**
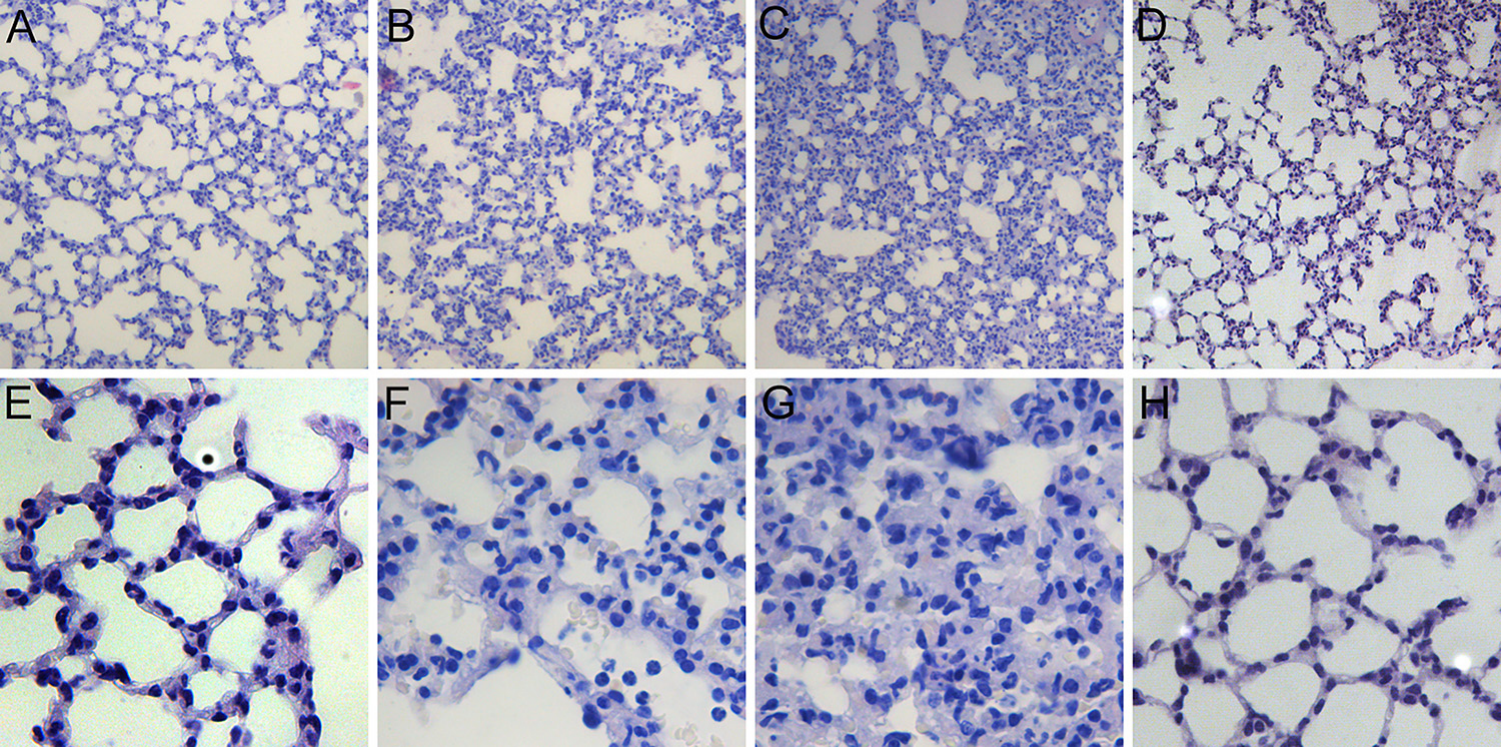
Pathological changes in lung tissue in mice after treatment. A&E) PBS, B&F) LPS, C&G) LPS + Cyclopamine, D&H) Cyclopamine. The imagines were from tissue sections taken at 12h after treatment. Magnitude: x100 for A, B, C, D; x400 for E, F, G, H.

The lung tissue sections stained with HE from each group of mice that were taken 12h after last injection are shown in Fig 1. Under the microscope, the LPS group showed diffuse lesions with extensive interstitial inflammatory cell infiltration, alveolar edema, and alveolar septa thickening (Fig 1B & 1F). The LPS-C group also demonstrated the same phenomena as the LPS group but more severely (Fig 1C & 1G). Another control of normal mice injected with cyclopamine (Fig 1D & 1H) showed normal alveolar septa without any inflammatory cell infiltration similar as the normal control (Fig 1A & 1E).

The differences of the thickness of alveolar septa in each group were shown in **S2 Table**. Both LPS and LPS-C groups exhibited significantly thickened alveolar septa in all time points compared with control group. Cyclopamine group showed that the cyclopamine alone has no measurable effect on the lung tissue (**S2 Table**).

The extent of lung injury was analyzed by scoring the tissue in a double blind examination for the following aspects: pulmonary edema, inflammatory infiltration, hemorrhage, atelectasis, and hyaline membrane formation. The combined injury scores for each group at 6h, 12h, and 24h after administration of PBS, LPS, LPS with cyclopamine or cyclopamine are listed in **S3 Table**. A significantly higher score was registered in the LPS-injected group (6h: 6.93±0.87 *vs*. 1.62±0.48, 12h: 5.72±0.61 *vs*. 1.49±0.53, 24h: 4.37±0.52 *vs*. 1.55±0.72; P<0.01) compared to that of the control at each time point. Administration of cyclopamine caused further injury in the lung; the injury scores were significantly higher compared with that of the group receiving LPS only (6h: 8.65±0.56 *vs*. 6.93±0.87, 12h: 7.23±0.63 *vs*. 5.72±0.61, 24h: 5.84±0.75 *vs*. 4.37±0.52; P <0.05, **S3 Table**). The injury score of the cyclopamine alone group showed no significant difference compared to the PBS group. It was also noteworthy that the injury scores in both LPS and LPS-C groups decreased over time.

The W/D ratio reflects the amount of extravascular lung water, which can be caused by the leakage of fluid into the extravascular space during the early phase of respiratory distress syndrome. A significantly higher W/D ratio of lung tissue was found in the LPS-injected mice at each time point compared with the control (P<0.01). When cyclopamine was used, the ratio of W/D became even higher (P<0.05, **S4 Table**). Both PBS and cyclopamine alone had no effect on the W/D ratio.

### Expression of the pro-inflammatory factor TNF-α

**Fig 2:**
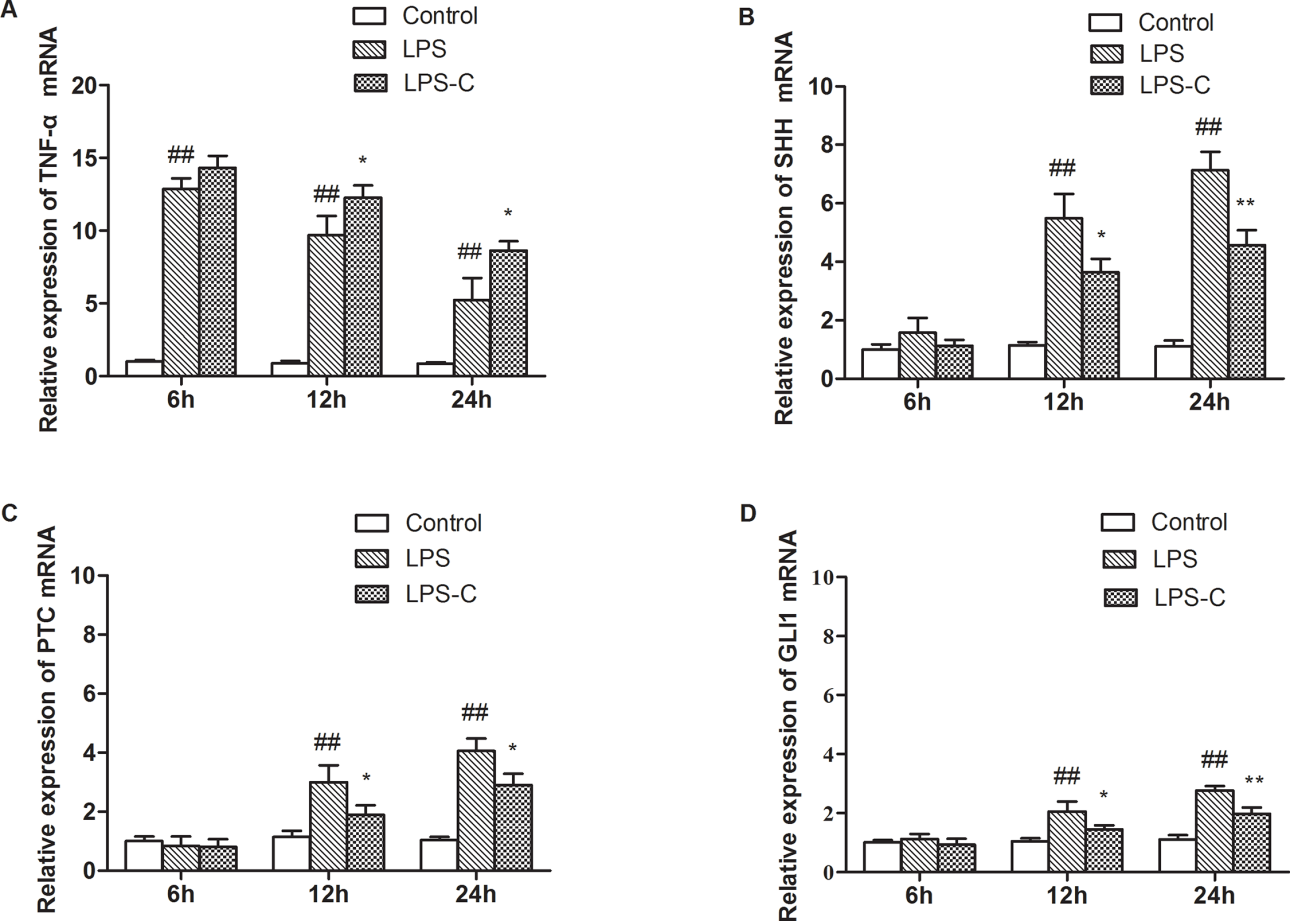
mRNA expression of TNF-α, SHH, PTC, and GLI1 from mouse lung tissue. mRNA expression at various time points from different treatment groups. Mice were grouped randomly, and were administered PBS, LPS, or LPS + cyclopamine. *P<0.05, **P<0.01 *vs*. LPS: ^##^P<0.01 *vs*. control.

TNF-α is a critical pro-inflammatory cytokine involved in the acute phase reaction. Its mRNA expression level was found to be elevated in the lung tissue of the LPS treated mice compared to the control mice (P<0.01) at all time-points examined. The mRNA expression level quickly peaked at the 6h time point and then gradually declined over time. A similar pattern was found after LPS and cyclopamine intervention; however, the expression level of TNF-α mRNA in the lung tissue of the LPS-C group were significantly higher than those of the LPS treatment alone (P<0.05, Fig 2A).

### Expression of the SHH signaling pathway

The SHH signaling pathway has been implicated in the acute lung injury response. The expression of SHH, PTC, and GLI1 mRNA levels in lung tissue were examined. At 6h after LPS administration, the SHH mRNA level showed a limited increase; this increase became more obvious at 12h, and even higher at 24h. The differences of expression levels seen in the LPS group and control at 12h and 24h time points were statistically significant (P<0.01). This increase was attenuated in mice receiving cyclopamine in addition to LPS (Fig 2B). The expression levels of the SHH receptor PTC and the downstream transcription factor GLI1 were also examined. No significant changes were found in the expression levels of PTC or GL1 mRNA at 6h in all groups. They were elevated starting from the 12h time point and increased further at 24h in the LPS and LPS-C groups. Similar to what was found in the changes of SHH expression levels, the increases of PTC and GLI1 mRNA expression levels were significantly attenuated after cyclopamine intervention (P<0.05 or P<0.01, Fig 2C and 2D).

**Fig 3:**
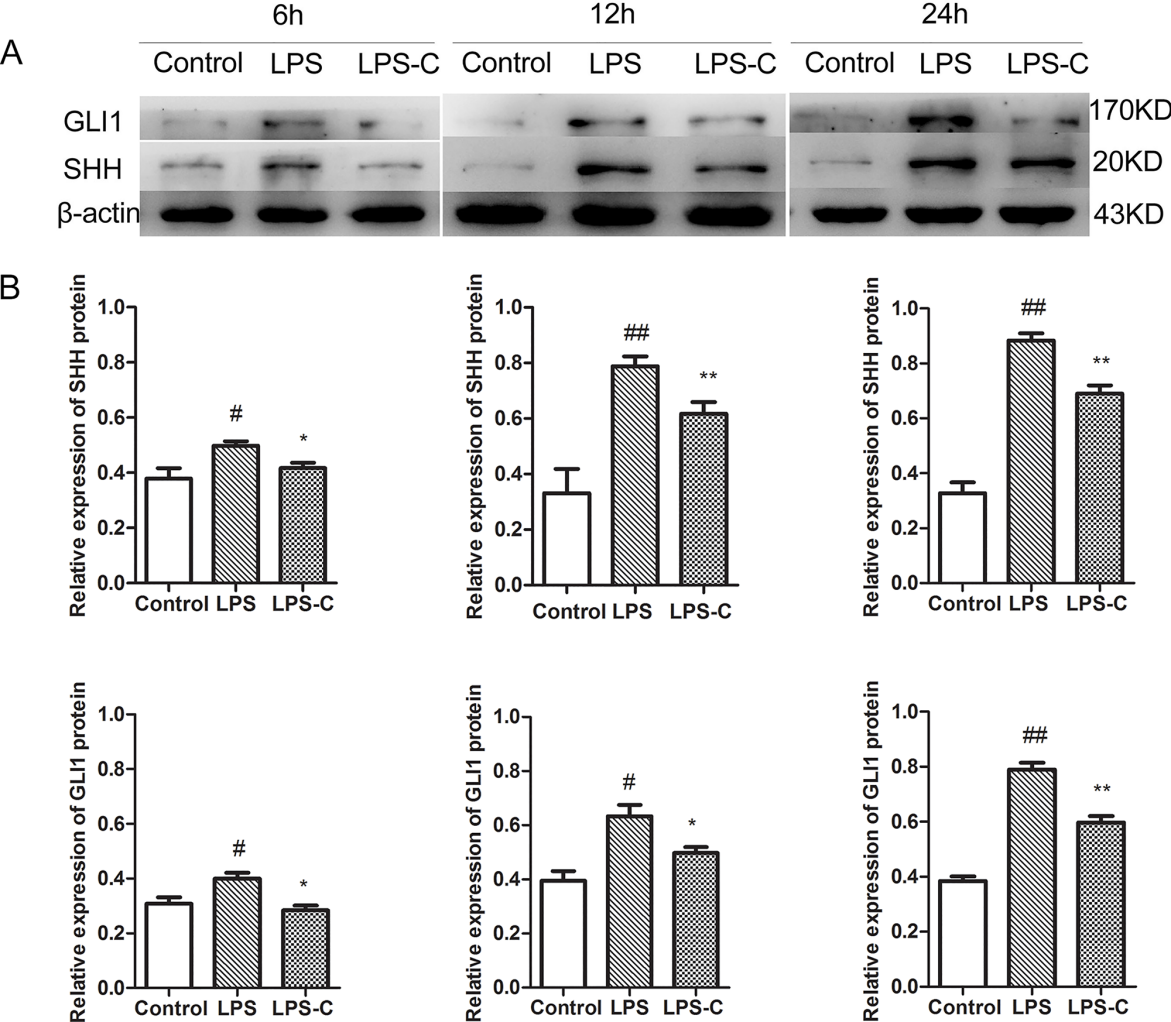
SHH and GLI1 protein production in lungs at various time points after treatment. Panel A: Western blot for protein production; Panel B: semi-quantitative analysis by densitometry; β-actin was used as an internal reference. *P<0.05, **P<0.01 *vs*. LPS treated; ^#^P<0.05; ^##^P<0.01 *vs*. control.

The protein levels of both SHH and GLI1 were further studied in the lung tissue via western blot analysis. Mice receiving LPS injection demonstrated significantly elevated levels of SHH and GLI1 protein in the lung tissue in a time-dependent manner over the control, in which levels of both proteins became elevated at 6h and increased further during the time course of examination (P<0.01, Fig 3). The increases on both SHH and GLI1 protein levels at 6h time point were not accompanied with increase of mRNA levels, suggesting that the response to the injury may have stabilized both SHH and GLI1 protein. After the application of cyclopamine, both SHH and GLI1 protein expression were significantly reduced comparing to the LPS only group (Fig 3).

## Discussion

The SHH signaling pathway is an essential component for normal embryonic growth and development. It is also shown to play important roles in adult tissue maintenance and regeneration. In this study, we examined the role of SHH signaling pathway in an endotoxin induced ALI mouse model. We found that while the expression of the SHH pathway components (SHH, PTC, and GLI1) in either mRNA or protein levels were low in the lung tissue of normal mice, their expression was greatly increased in the LPS induced ALI mice, indicating an activation of the SHH signaling pathway in response to ALI in this animal model. We also found that the alleviation of the lung injury induced by LPS was correlated with the increased expression of SHH pathway elements. Both the combined lung tissue injury scores and the expression levels of pro-inflammatory cytokine TNF-α started to decline at 12h after LPS injection when the expressions of SHH pathway components became significantly higher.

This finding was further confirmed by the application of the SHH antagonist cyclopamine. Cyclopamine has been used as an SHH antagonist in many studies [20,21]. When cyclopamine was administered to LPS challenged mice, we observed significant decreases in the expression of SHH, PTC, and GLI1. This was accompanied by increases in TNF-α mRNA expression, lung tissue injury score, and the lung W/D weight ratio. These findings strongly suggested that SHH signaling pathway activation was correlated with the reduced degree of lung injury. This protective mechanism was interrupted when SHH signaling was inhibited.

It is well known that the natural occurred alkaloid cyclopamine binds to SMO and induce SMO misfolding within the endoplasmic reticulum [22]. In this study, our results showed a decreased mRNA level of the SHH signaling pathway including SHH, PTC, and GLI1, suggesting that application of cyclopamine also had inhibitive effect on the expression of these proteins. SHH signaling can be in a paracrine or autocrine fashion. One of the possible mechanisms could be the block of downstream signaling of SHH also has impact in SHH expression.

Our finding is consistent with reports about the role that the SHH signaling pathway plays in tissue development and repair [23,24]. A huge volume of studies on the SHH signaling pathway have established its critical function relative to embryonic development. It can induce pulmonary branch formation and epithelial—mesenchymal transition [25], direct differentiation of mesoderm in pancreas [26], and regulate epithelial differentiation in prostate development [27]. SHH signaling is critical for the specification and differentiation of neural stem cells [28] and the osteoblast lineage [29]. Recent studies found the pathway being activated in many physiological and pathological processes beyond the embryonic stage. The best example is the liver regeneration process. The SHH pathway plays a major role in liver repair and regeneration in adults [30,31]. Blocking of SHH signaling inhibits portal tract expansion, accumulation of liver progenitors, and liver cell proliferation [30].Its activation has also been found in hyperoxia and bleomycin induced lung injury [25,32] although the mechanism is not clear yet. Renault *et al*. found that activation of the SHH pathway can promote angiogenesis by myogenesis, which could be a possible mechanism for SHH function [33]. SHH induces overexpression of several pro-angiogenic growth factors, including VEGFA and angiopoietin-1 (Ang1) [34,35].SHH has also been reported to activate the phosphatidylinositol 3-kinase (PI3-K)/Akt pathway [36], to further enhance production of nitric oxide (NO), which plays a key role in cardiovascular protection.

Using the LPS-induced ALI mouse model, we demonstrated that the activity of SHH pathway was associated with lung repair in ALI mouse model. However, this study did not exclude the possibility that there are other components involved in the lung repair process. It is only reasonable to assume that the process would require multiple signaling pathways and factors. Certain limitations existed in this study, such as that a functional assay of GLI1 would be significant to verify the functional change of the SHH pathway, and a statistical correlation analysis for the activation of the SHH signaling pathway and alleviation of lung injury would provide more convincing evidence for the relationship between them. More study is needed to identify the downstream target of the SHH pathway in the repair process in order to better understand its mechanism. Nevertheless, the discovery of the function of the SHH signaling pathway in endotoxin induced ALI has an immediate implication that the protective mechanisms might be taken into consideration in drug development.

## Supporting Information

S1 Table Primer sequences used for RT-PCR. (DOCX)

S2 Table The measurement of alveolar septa in mice of different groups. (DOCX)

S3 Table Pathological score for lung tissue injury (mean±SEM, n = 5 in each time point of each group). (DOCX)

S4 Table Weight ratio of lung tissue under wet and dry (W/D) condition (mean±SEM, n = 5 in each time point of each group). (DOCX)
