# Supplementary material for: Sonic Hedgehog Signaling: Evidence for Its Protective Role in Endotoxin Induced Acute Lung Injury in Mouse Model
Source: PLoS One. 2015 Nov 6;10(11):e0140886. doi: 10.1371/journal.pone.0140886 (PMC4636314; doi:10.1371/journal.pone.0140886)
Supplement: S1 Table — (DOCX) [file pone.0140886.s001.docx]

**Table 1.** **Primer sequences used for RT-PCR.**

| **Primers** | **Forward** | **Reverse** | **Product** |
| --- | --- | --- | --- |
| **TNF-α** | 5’-CCCTTTACTCTGACCCCTTTATTGT-3’ | 5’-TGTCCCAGCATCTTGTGTTTCT-3’ | 143bp |
| **SHH** | 5’-CCAACGTAGCCGAGAAGACC-3’ | 5’-TCCCGTGTTTTCCTCATCCT-3’ | 134bp |
| **PTC** | 5’-ACACTTCAGGGGCTACGACTATG-3’ | 5’-TGGGGCGACACTTTGATG-3’ | 118bp |
| **GLI1** | 5’-TGAGGTGGGCAGGTTAGGA-3’ | 5’-CAGAGGGAGATGGGGTGTTTT-3’ | 194bp |
| **β-actin** | 5’-CATCCGTAAAGACCTCTATGCCAAC-3’ | 5’-ATGGAGCCACCGATCCACA-3’ | 171bp |
