# Supplementary material for: Sonic Hedgehog Signaling: Evidence for Its Protective Role in Endotoxin Induced Acute Lung Injury in Mouse Model
Source: PLoS One. 2015 Nov 6;10(11):e0140886. doi: 10.1371/journal.pone.0140886 (PMC4636314; doi:10.1371/journal.pone.0140886)
Supplement: S2 Table — (DOCX) [file pone.0140886.s002.docx]

**Table 2.** **The measurement of alveolar septa in mice of different groups.**

|  | **6h (μm)** | **12h (μm)** | **24h (μm)** |
| --- | --- | --- | --- |
| **Control** | 6.25±1.37 | 7.29±1.89 | 6.60±1.92 |
| **LPS** | 24.36±5.99^**^ | 20.12±6.65^*^ | 19.73±4.87^*^ |
| **LPS-C** | 37.61±5.12^#^ | 34.58±5.93^#^ | 32.25±4.91^#^ |
| **C** | 7.48±2.52 | 7.50±2.09 | 6.53±1.98 |

*P<0.05 *vs.* control; **P<0.01 *vs.* control; ^#^P<0.05 *vs.* LPS

LPS: lipopolysaccharides, LPS-C: lipopolysaccharides-cyclopamine; C: cyclopamine.

Five mice from each group were tested.
