# Supplementary material for: Sonic Hedgehog Signaling: Evidence for Its Protective Role in Endotoxin Induced Acute Lung Injury in Mouse Model
Source: PLoS One. 2015 Nov 6;10(11):e0140886. doi: 10.1371/journal.pone.0140886 (PMC4636314; doi:10.1371/journal.pone.0140886)
Supplement: S3 Table — (DOCX) [file pone.0140886.s003.docx]

**Table 3.** **Pathological score for lung tissue injury (mean*±*SEM*,* n=5 in each time point of each group).**

|  | **6h** | **12h** | **24h** |
| --- | --- | --- | --- |
| **Control** | 1.62±0.48 | 1.49±0.53 | 1.55±0.72 |
| **LPS** | 6.93±0.87^**^ | 5.72±0.61^**^ | 4.37±0.52^**^ |
| **LPS-C** | 8.65±0.56^*^ | 7.23±0.63^*^ | 5.84±0.75^*^ |
| **C** | 1.78±0.52 | 1.58±0.63 | 1.51±0.49 |

*P<0.05 *vs.* LPS; **P<0.01 *vs.* control

LPS: lipopolysaccharides, LPS-C: lipopolysaccharides-cyclopamine; C: cyclopamine.

Five mice from each group were evaluated.
