# Supplementary material for: Sonic Hedgehog Signaling: Evidence for Its Protective Role in Endotoxin Induced Acute Lung Injury in Mouse Model
Source: PLoS One. 2015 Nov 6;10(11):e0140886. doi: 10.1371/journal.pone.0140886 (PMC4636314; doi:10.1371/journal.pone.0140886)
Supplement: S4 Table — (DOCX) [file pone.0140886.s004.docx]

**Table 4.** **Weight ratio of lung tissue under wet and dry (W/D) condition (mean*±*SEM, n=5 in each time point of each group).**

|  | **6h** | **12h** | **24h** |
| --- | --- | --- | --- |
| **Control** | 4.12±0.18 | 4.07±0.06 | 4.05±0.08 |
| **LPS** | 4.98±0.12^**^ | 4.71±0.08^**^ | 4.48±0.14^#^ |
| **LPS-C** | 5.24±0.07^*^ | 4.93±0.09^*^ | 4.79±0.12^*^ |
| **C** | 4.20±0.11 | 4.11±0.14 | 4.08±0.09 |

*P<0.05 *vs.* LPS; **P<0.01, ^#^P<0.05 *vs.* control
